# Gulf War Illness and Inflammation: Association of symptom severity with C-reactive protein

**DOI:** 10.29245/2572.942x/2019/2.1245

**Published:** 2019-04-10

**Authors:** Lisa M. James, Brian E. Engdahl, Rachel A. Johnson, Apostolos P. Georgopoulos

**Affiliations:** 1 Brain Sciences Center, Department of Veterans Affairs Health Care System, Minneapolis, MN, 5541, USA; 2 Department of Neuroscience, University of Minnesota Medical School, Minneapolis, MN 55455, USA; 3 Department of Psychiatry, University of Minnesota Medical School, Minneapolis, MN 55455, USA; 4 Department of Psychology, University of Minnesota Medical School, Minneapolis, MN 55455, USA; 5 Department of Neurology, University of Minnesota Medical School, Minneapolis, MN 55455, USA

**Keywords:** Gulf War Illness, Inflammation, C-reactive protein, Neuroimmune, Persistent Antigens, Immunity

## Abstract

Gulf War Illness (GWI) is a chronic multi-system condition that has affected one-third of U.S. veterans who served in the Persian Gulf. Although GWI etiology remains unclear, mounting evidence points to immune system involvement and inflammation, in particular, as underlying the host of symptoms associated with the condition. Here we investigated the association between GWI symptoms and C-reactive protein (CRP), a marker of inflammation, in 76 veterans with GWI. Results indicated a highly significant positive association between CRP and mean GWI symptom severity. At the symptom domain level, CRP was significantly and positively associated with Pain, Neurocognitive/Mood, Fatigue, and Respiratory symptom severity but not with Skin or Gastrointestinal symptom severity. These results support the premise that GWI symptoms, particularly those implicating brain involvement, are a result of neuroinflammation. The cause for inflammation is not known. We have hypothesized that at the root of GWI are harmful persistent antigens stemming from environmental exposures associated with service during the Gulf War that could not be successfully eliminated due to lack of specific immunity^1,2^. Work is underway in our laboratory to identify and eliminate persistent antigens in veterans with GWI which we anticipate will result in reduced inflammation and reduced GWI symptoms.

## Introduction

Gulf War Illness is a chronic disease of unclear etiology that has affected a large number of veterans of the 1990-1991 Persian Gulf War. Symptoms affect several systems and include fatigue, musculoskeletal pain, neurological and cognitive impairment, and mood disruptions^3^ in addition to respiratory, gastrointestinal, and dermatological complaints^4^. Burgeoning evidence suggests that genetic vulnerability related to immune system functioning coupled with environmental insults may underlie the host of symptoms observed in GWI^1,2^.

Increasingly, immune system disruption has been recognized in relation to GWI^1,5-14^. Consistent with evidence of immune-mediated loss of blood brain barrier integrity^15^, alterations in brain structure and function have been associated with GWI^16-19^. Furthermore, brain function in GWI has been shown to be indistinguishable from that of known immune-related conditions^20^. Robust evidence of immune involvement in conjunction with brain alterations suggests GWI is best characterized as a neuroimmune condition^7,20^ resulting from persistent antigens that contribute to immune system disruption and inflammation^2^.

Evidence of inflammation has been reported in veterans with GWI^10,21,22^. For example, C-reactive protein (CRP), a non-specific acutephase biomarker of inflammation, has been shown to be elevated in GWI^10,21^. In fact, of 61 plasma proteins evaluated in one study of GWI^10^, CRP was one of only 6 that significantly differed between veterans with and without GWI and the only protein identified from a stepwise multivariate logistic regression model that contributed to a diagnostic model of GWI (along with lymphocytes, and monocytes). CRP is synthesized primarily in the liver hepatocytes; however, other cell types including smooth muscle cells, macrophages, endothelial cells, lymphocytes, and adipocytes have also been shown to synthesize CRP^23^. Notably, emerging evidence also indicates local CRP production in human neuronal cells of patients with Alzheimer’s disease and upregulation of CRP in Alzheimer’s-affected brain areas^24^. Finally, CRP has historically been viewed as a marker of inflammation that arises in response to inflammatory cytokines such as interleukin 6; yet, mounting evidence suggests CRP may also play a causal role in inflammation^23^. Thus, CRP may both signal and potentiate inflammation in various cell types and tissues, perhaps underlying the diffuse symptoms involving multiple systems as seen in GWI. To date, however, relatively little is known about CRP as it relates to GWI. Here we aim to evaluate the association between GWI symptoms and CRP levels to further assess the link between CRP and GWI symptomatology.

## Materials and Methods

### Participants:

A total of 76 veterans with GWI and no comorbidities were studied (70 men, age 56.3 ± 8.1 y [mean ± SD], 6 women, age 50.5 ± 5.2 y). GWI status was determined using a self-report symptom checklist that evaluates the presence and severity of various symptoms comprising 6 domains characteristic of GWI: fatigue, pain, neurological/mood/cognitive, gastrointestinal, skin rashes, and respiratory. Items were rated on a scale from 0 (absent) to 3 (severe). Veterans who met either Center for Disease Control^1^ or Kansas criteria^2^ for GWI were included in the present analyses. All study protocols were approved by the appropriate Institutional Review Boards. Study participants provided informed consent, in adherence to the Declaration of Helsinki, and were financially compensated for their time.

### CRP:

Non-fasting peripheral venous blood samples were collected for evaluation of high sensitivity C reactive protein and analyzed using standard procedures by the Minneapolis VAHCS Clinical Laboratory.

### Other variables:

The Body Mass Index (BMI) was 31.65 ± 5.21 (mean ± SD, N = 76). No participant reported illegal drug use or alcohol abuse. Twenty-one participants were receiving medications (15 on antidepressants, 4 on pain relievers, 2 on beta blockers); five were receiving opioids for pain relief (3 tramadol, 2 hydrocodone).

### Data analysis:

The IBM-SPSS statistical package (version 23) was used to analyze the data. The average GWI symptom severity across all domains was computed as well as the average symptom severity within each of the 6 GWI symptom domains. The correspondence between GWI symptoms and CRP were evaluated using stepwise linear regressions., where GWI symptom severity was the dependent variable, CRP was the independent variable, and medication status and BMI were covariates.

## Results

### CRP.

**Figure 1:**
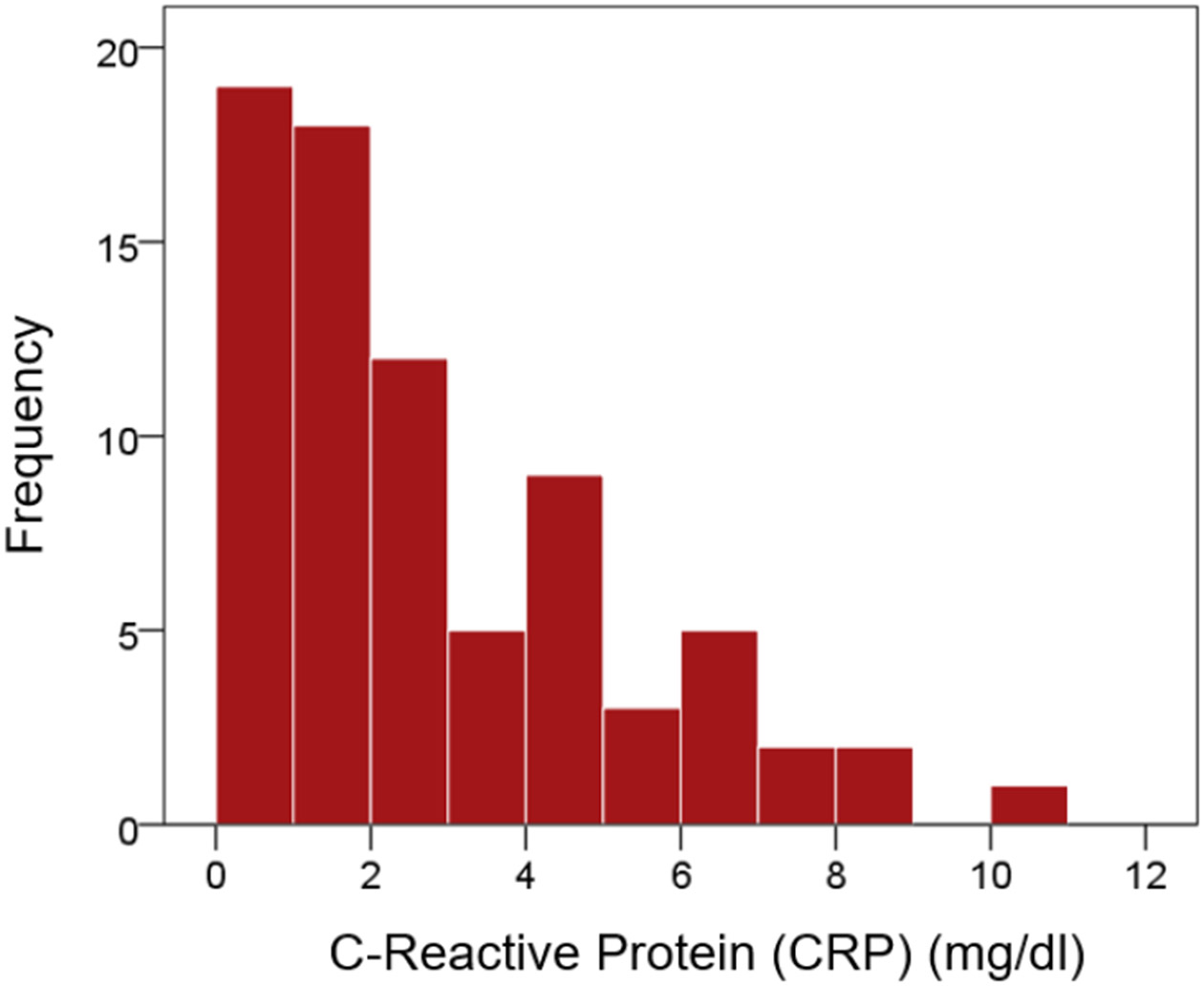
Exponential distribution of CRP values.

**Figure 2:**
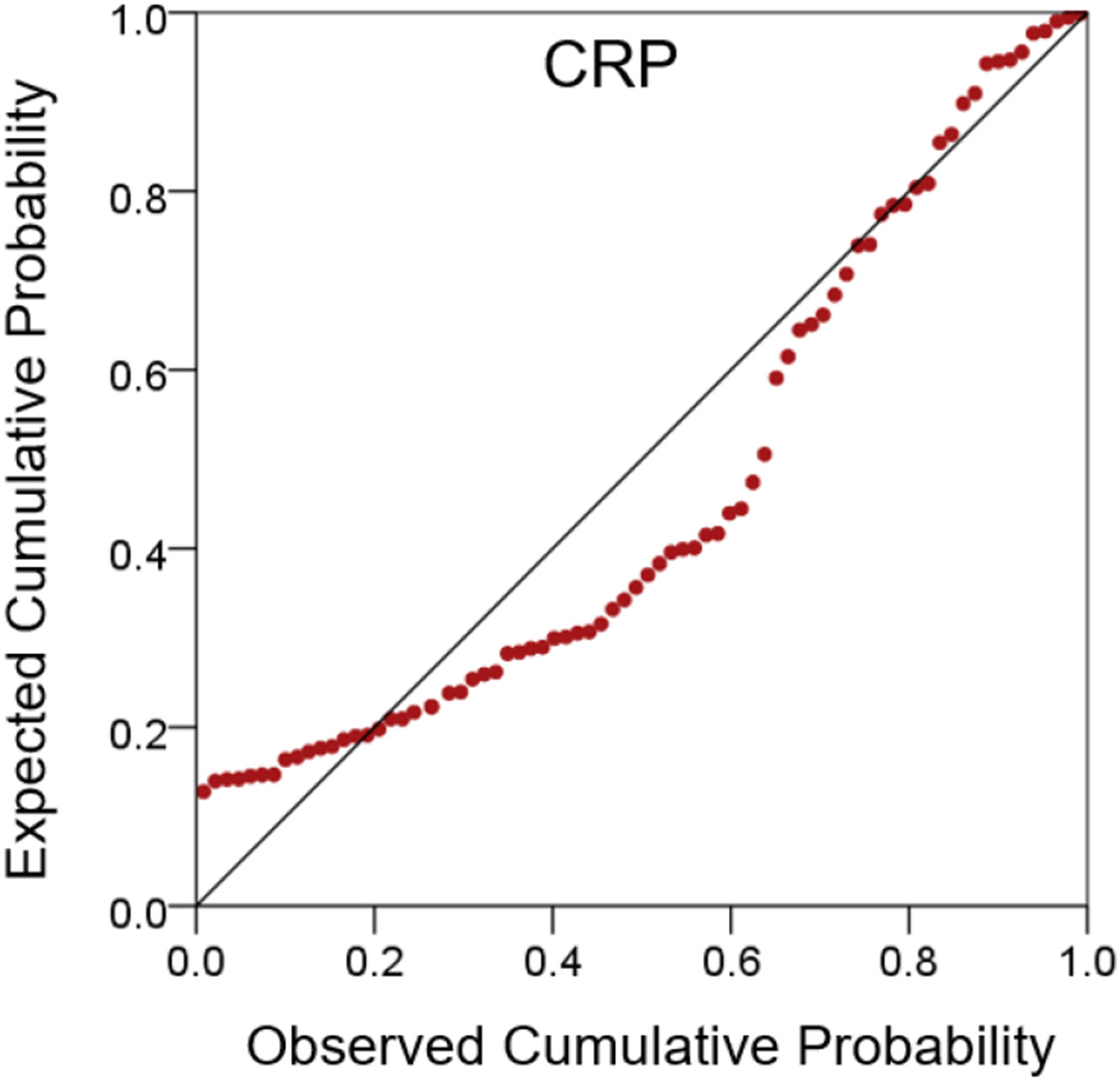
Probability plot of CRP values, under the assumption of a normal distribution. A substantial deviation from normality is indicated by the deviation of the plotted values from the midline.

**Figure 3:**
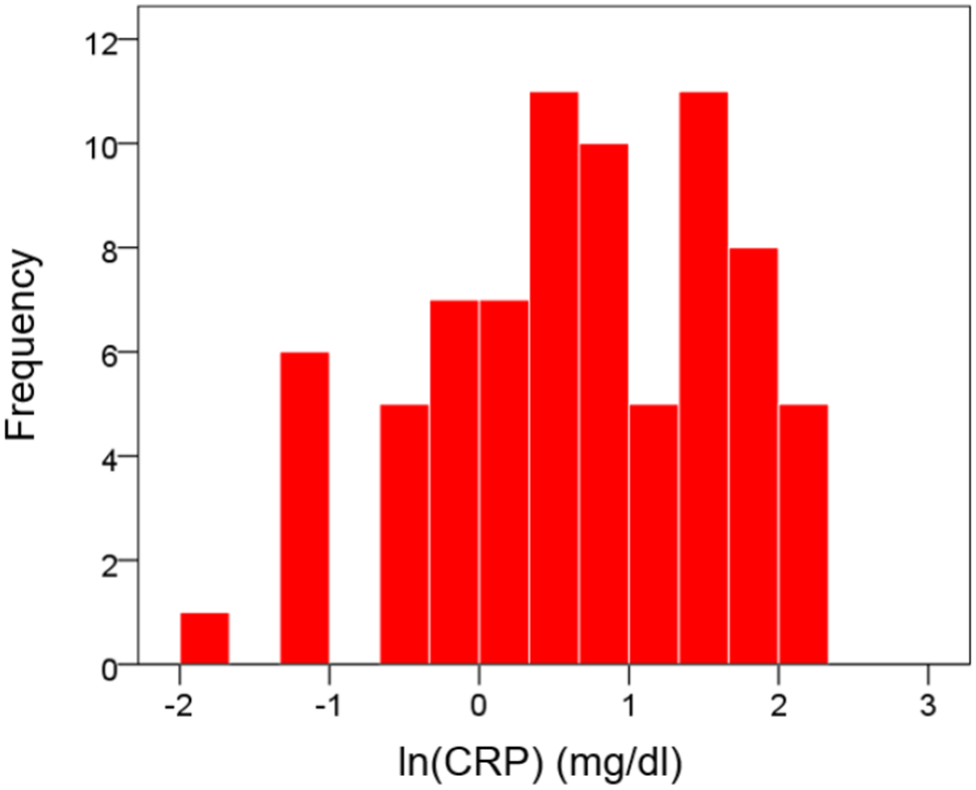
Log-transformed distribution of CRP values.

**Figure 4:**
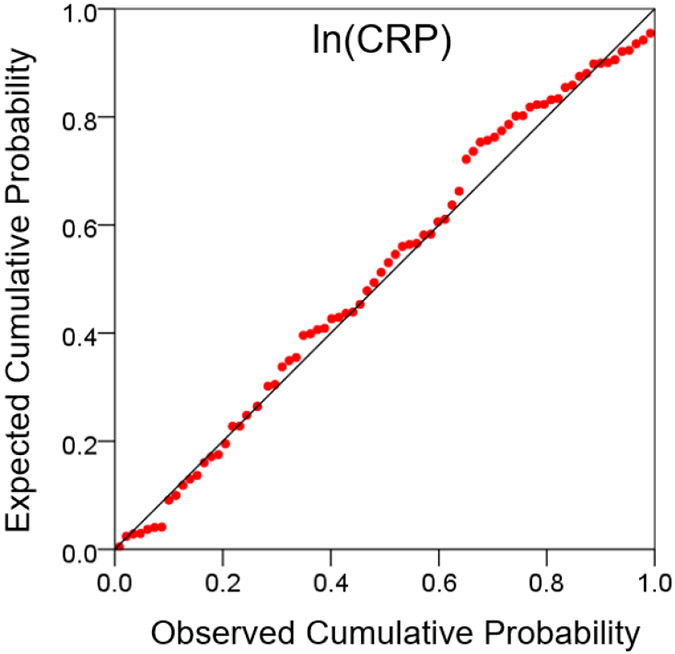
Probability plot of log-transformed CRP values, under the assumption of normal distribution. Notice the closeness of the plotted values to the midline.

CRP values were distributed in a non-normal fashion (Figures 1 and 2). Therefore, they were transformed to their logarithms to normalize their distribution (Figures 3 and 4). The log-transformed CRP values, $CRP^{\prime }$, were used in all subsequent analyses:

(1) $$CRP^{\prime }=\text{ln}(CRP)$$

### Association of GWI symptom severity with CRP.

**Figure 5:**
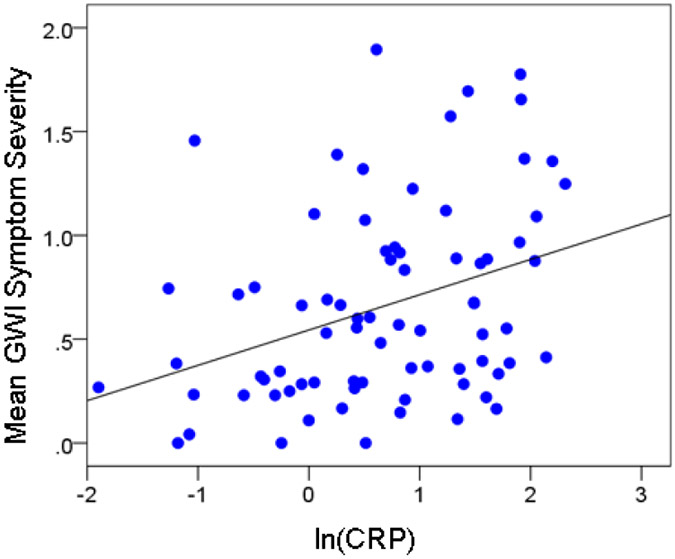
Association of mean GWI symptom severity with log-transformed CRP.

The mean of GWI symptom severity (across the 6 GWI symptom domains) was significantly and positively associated with ln(CRP) [$CRP^{\prime }$, equation 1] (Figure 5; r = 0.353, P = 0.002). The results of the stepwise regression analyses are shown in Table 1.

It can be seen that $CRP^{\prime }$ had a significant effect in all but the Gastrointestinal and Skin domains. BMI did not have a significant effect in any analysis, whereas medication status had a significant effect in all but the Respiratory domain.

## Discussion

Here we investigated the association between inflammation and GWI symptoms in a sample of GW veterans and found a highly significant positive association between CRP, a marker of inflammation, and GWI symptom severity. The results add to the literature highlighting the role of inflammation in GWI^10^ and point to the potential benefit of interventions for GWI aimed at reducing inflammation.

GWI is a chronic condition characterized by widespread symptoms spanning several systems including the central nervous system, respiratory, dermatologic, and gastrointestinal system. Of these, the brain is prominently involved with three of the six symptom domains - fatigue, pain, and neurocognitive/mood symptoms - implicating brain involvement. Notably, all three of these domains were highly significantly associated with CRP in the present study, further cementing GWI as a neuroimmune condition^20^. Respiratory symptoms were the only other domain that was significantly associated with inflammation.

Based on a series of recent findings in our lab we have proposed that GWI is a result of persistent antigens stemming from environmental exposures associated with service during the Gulf War that could not be successfully eliminated due to lack of specific immunity^25^. Initial support of the “Persistent Antigen” hypothesis was established on findings demonstrating that 6 Class II human leukocyte antigens (HLA) distinguish healthy Gulf War veterans from veterans with GWI^1^. Specifically, the 6 alleles were significantly more common among healthy veterans suggesting that their presence is protective against GWI; conversely, the absence of these HLA alleles and consequent lack of protection results in GWI. Subsequently, we demonstrated that one of the 6 protective alleles, in particular – HLADRB1*13:02 – is highly protective against brain atrophy^2,26^ that has been observed in GWI veterans^16^. The protection provided by the presence of these class II alleles is inherent in their function which is elimination of foreign antigens via antibody production; however, the ability to stimulate antibody production hinges on a match between HLA proteins and epitopes derived from foreign antigens. In the absence of a match, the antigen persists, resulting in inflammation (reflected here by elevated CRP) and other detrimental effects including cell damage and atrophy. Thus, we suspect that prominent GWI symptoms, particularly those implicating brain involvement, are a result of neuroinflammation due to the persistence of foreign antigens resulting from lack of HLA protection.

Although the specific cause of inflammation in GWI veterans remains to be elucidated, we hypothesize that it is the result of the existence of harmful persistent antigens in GWI; indeed, we have studies underway aimed at identifying persistent antigens in veterans with GWI with the goal of ultimately eliminating them (and thereby reducing inflammation) via personalized immunotherapy. Two recent in vitro studies in our lab have provided initial evidence supporting immunotherapy as a promising intervention for GWI. Specifically, we have demonstrated that serum from veterans with GWI results in detrimental changes to cell morphology in neural cultures; however, the addition of serum from healthy Gulf War veterans^27^ or human immunoglobulin G (IgG)^28^ neutralize those damaging effects. The neutralizing effects are presumed to result from the ability of antibodies present in serum from healthy Gulf War veterans and in pooled IgG to eliminate persistent antigens in veterans with GWI. We anticipate that elimination of persistent antigens would result in reduced inflammation and reduced GWI symptoms. Inflammatory response regulation via monoclonal antibodies targeting specific cytokines or neuroendocrine control of the cytokine network, as is under investigation in other diseases^29^ may prove to be useful alternative therapeutic strategies for reducing GWI-related inflammation and symptoms.

## Figures and Tables

**Table 1.** Result of the stepwise regression analyses. P-values are those obtained for the final-step model; “excluded” denotes absence of the variable from this model.

Dependent variable (symptom score)	Independent variable	Covariates	
GWI symptom domain	$CRP^{\prime }$	Medication status	BMI
Mean symptom severity	P < 0.001	P < 0.001	P = 0.242 (excluded)
Fatigue	P < 0.001	P < 0.001	P = 0.838 (excluded)
Pain	P = 0.013	P = 0.024	P = 0.163 (excluded)
Neurocognitive	P = 0.004	P < 0.001	P = 0.977 (excluded)
Respiratory	P = 0.018	P = 0.317 (excluded)	P = 0.618 (excluded)
Gastrointestinal	P = 0.097 (excluded)	P = 0.012	P = 0.112 (excluded)
Skin	P = 0.603 (excluded)	P = 0.045	P = 0.323 (excluded)
